# Current Progress and Future Trends in Carbon Sources and Sinks in Farmland Ecosystems: A Bibliometric Analysis (2002–2023)

**DOI:** 10.3390/biology14040365

**Published:** 2025-04-02

**Authors:** Yugong Pang, Menghao Zhang, Hesen Zhong, Tibihenda Cevin, Chuanzhun Sun, Shoutao Zhang, Xinyu Li, Jun Dai, Chengshuai Liu, Chi Zhang

**Affiliations:** 1 College of Natural Resources and Environment, South China Agricultural University, Guangzhou 510642, China; pangyugong1123@163.com (Y.P.); 15737316382@163.com (M.Z.); 15775071468@163.com (H.Z.); kevootibe@gmail.com (T.C.); 19953345795@163.com (S.Z.); lxinyu224gd@163.com (X.L.); jundai@scau.edu.cn (J.D.); csliu@scau.edu.cn (C.L.); 2 Tanzania Agricultural Research Institute, Dodoma 1571, Tanzania; 3 College of Public Management, South China Agricultural University, Guangzhou 510642, China; suncz@scau.edu.cn

**Keywords:** farmland ecosystem, carbon sources, carbon sink, carbon sequestration, soil organic carbon (SOC), bibliometric analysis

## Abstract

Farmland ecosystems, as the most active carbon pool, are integral to global climate change and carbon cycling. Therefore, systematically studying the roles of carbon sources and sinks in farmland ecosystems is essential to deepening our understanding of the carbon cycle and meeting the goals of “peak carbon emissions” and “carbon neutrality” in agriculture. Using the Web of Science database, this study reviewed 1411 articles (2002–2023) via bibliometric analysis to identify key research themes, trends, future priorities and address suggestions for future directions in farmland ecosystem carbon sources and sinks. The main results include the following: (1) Over the past 22 years, global research in this field has shown a consistent growth trend, with remarkably rapid expansion in the past three years. China, the United States, and Germany are the most influential countries. As the research scope has expanded, the field has evolved into an interdisciplinary domain. (2) The diversity of this research area has become enriched, and the research content is becoming more refined and systematic. The main research topics focus on carbon sequestration, soil organic carbon (SOC), farmland management, greenhouse gas (GHG) emissions, carbon stocks, ecosystem services, land use changes, climate change, and spatiotemporal heterogeneity. (3) Current research hotspots primarily focus on studying soil microbial carbon sequestration mechanisms, the application of remote sensing technologies, and reducing GHG emissions to achieve “carbon neutrality”. While existing studies have systematically elucidated carbon sequestration mechanisms mediated by soil aggregates, microorganisms, and minerals, critical knowledge gaps persist. Regional disparities in the relative contributions of these mechanisms remain unresolved, compounded by methodological inconsistencies in carbon assessment that introduce substantial uncertainties. Although farmland management practices are identified as pivotal drivers of carbon flux variation, the interactive effects of anthropogenic interventions and natural factors on ecosystem-scale carbon balance require further mechanistic exploration. This review provides a comprehensive reference for further study on carbon sources and sinks of farmland ecosystems and devising effective emission reduction strategies.

## Simple Summary

Farmland ecosystems play a vital role in global carbon cycling and climate change, yet understanding how these systems balance carbon absorption and emissions remains challenging. This study reviewed 1411 research articles (2002–2023) to identify key findings and challenges in this field. Research has grown steadily over the past two decades, with China, the U.S., and Germany leading global efforts. Recent advances show that soil microbes are central to locking carbon into soils. In contrast, science-based practices like conservation tillage can significantly boost carbon storage. Scientists have also focused on reducing greenhouse gases from farming and using satellite technology to track carbon changes. However, regional differences in carbon processes and inconsistent methods to estimate carbon sources versus sinks create uncertainties. For example, some regions naturally store more carbon, but existing frameworks struggle to explain these variations. Therefore, future research should deeply explore the complex interactions within soil carbon sequestration mechanisms in farmland ecosystems and optimize carbon accounting methods. By clarifying these mechanisms, our findings provide crucial theoretical support for global strategies to achieve agricultural “carbon neutrality”, ensuring farmland can mitigate climate change while sustainably producing food.

## 1. Introduction

With the global economy shifting towards achieving net-zero greenhouse gas (GHG) emissions, reducing GHG emissions has emerged as one of the most critical challenges confronting the agricultural sector [1,2]. Farmland ecosystems account for approximately 12% of the global land area and play a dual role as atmospheric carbon sources and sinks [3]. On one hand, farmland-related agricultural activities contribute approximately 23% to 30% of global GHG emissions [4]. Moreover, global farmland carbon stocks range from 128 to 165 Pg C, representing 8% to 10% of the global carbon stock [5,6]. Regardless of their role as a source or sink of GHG, farmland ecosystems significantly impact atmospheric CO_2_ concentrations [7,8]. Controlling GHG emissions from farmland and enhancing carbon sequestration in these ecosystems are effective strategies for mitigating climate change and achieving the goals of “peak carbon” and “carbon neutrality”.

Scholars have contributed significantly to advancing our understanding of carbon sources and sinks in farmland ecosystems. In examining the carbon sequestration effects and potential of farmland management practices, researchers have explored the impacts of various tillage practices, fertilization methods, and water management patterns on carbon sequestration and emission reduction in farmland soils. West et al. employed a full-carbon-cycle analysis methodology to compare conventional versus no-tillage practices, showing that no-tillage practices offer more significant advantages in enhancing carbon sequestration and reducing emissions [9]. Similarly, Jiang et al. evaluated the impacts of varying nitrogen fertilizer application rates on carbon sequestration and emission reduction in single-season rice production through field experiments conducted in Zhejiang, China. They found that applying 225 kg∙ha^−1^ of nitrogen fertilizer in this region could simultaneously enhance grain yield, reduce GHG emissions, and increase system carbon sequestration [10]. Moreover, various methods have been developed to estimate carbon sources and sinks, including the emission factor method, life cycle method, and modeling techniques. Advancements in geographical information system (GIS) and remote sensing technology have facilitated the study of spatiotemporal variation of carbon sources and sinks within farmland ecosystems. For instance, Hei et al. used multi-source remote sensing data to reveal the spatiotemporal variation trends of net primary productivity (NPP) in farmland ecosystems [11]. In addition, some scholars have conducted reviews on carbon source and sink research in farmland ecosystems, but these are often limited to specific research directions or the progress within a specific country. For example, Li et al. conducted a comprehensive review of the uncertainties and discrepancies associated with estimating agricultural GHG emissions in China [12]. Similarly, Liu et al. reviewed GHG measurement methods, carbon source and sink assessment models for farmland ecosystems, as well as the key factors influencing the dynamics of carbon sources and sinks [13]. Nevertheless, their analysis of assessment methods was relatively limited, focusing solely on the emission factor method without providing comparative analyses of other approaches. Furthermore, Li et al. systematically reviewed accounting methods for carbon sources and sinks in farmland ecosystems [14]. Research on carbon sources and sinks in farmland ecosystems represents a global challenge that intersects with multiple disciplines. Focusing solely on specific aspects or studies from a single country makes it difficult to achieve a comprehensive understanding of existing research characteristics and emerging trends. Therefore, a thorough, detailed, and objective summary of existing research in this field is crucial for its future development.

Bibliometric analysis is a well-established method for quantitatively evaluating research progress and identifying developmental trends within specific fields of study [15,16]. This study applies bibliometric analysis to systematically review the literature on carbon sources and sinks in the farmland ecosystem, aiming to identify the existing knowledge domain, recognize emerging trends, and guide future research. This paper mainly addresses four primary objectives: (1) It aims to provide an overview of studies on carbon sources and sinks in farmland ecosystems published from 2002 to 2023. (2) It summarizes the main research topics and principal theories in this field. (3) It analyzes the current research hotspots and future development trends in this field. (4) It identifies existing research gaps and proposes recommendations for future studies.

## 2. Materials and Methods

**Figure 1:**
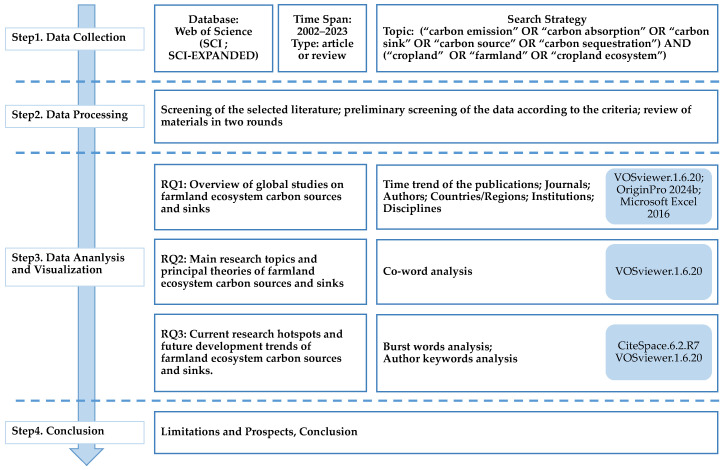
Research framework of this study.

In this study, we employed CiteSpace.6.2.R7 and VOSviewer.1.6.20 for bibliometric analysis. CiteSpace is a Java-based tool that supports in-depth data mining, knowledge mapping networks, and visualization of research evolution, including the exploration of emerging research hotspots and frontiers [15]. VOSviewer is a bibliometric tool for knowledge units from the literature with unique advantages in clustering techniques and network mapping visualization, useful for identifying central themes in scientific research in the following areas [17]. The data for this study were sourced from the Web of Science (WOS) database, restricted to the Web of Science Core Collection (WOSCC). Our search parameters in Web of Science were “Topic: (“carbon emission” OR “carbon absorption” OR “carbon sink” OR “carbon source” OR “carbon sequestration”) AND (“cropland” OR “farmland” OR “cropland ecosystem”)”, refined by the following: Document type: (Article or Review); Timespan: 2002–2023; Web of Science Index: Social Sciences Citation Index (SSCI) and Science Citation Index Expanded (SCI-EXPANDED). The search resulted in 1513 articles (Figure 1). All these articles were downloaded on 19 February 2024. To address the issue of irrelevant records from the database, a manual selection process was conducted in two rounds by one Master’s student and two PhD students (Figure 1). In the first round, the Master’s student eliminated articles irrelevant to our topic by reading the titles, abstracts, and keywords of 1513 articles. In the second round, the two PhD students read the full texts of the remaining articles and determined whether they were relevant to our topic before making further exclusions, resulting in 1411 valid documents as data for analysis (article: 1346; review: 65). The data were then imported into CiteSpace and VOSviewer software for the analysis and visualization of various indicators, while OriginPro 2024b and Microsoft Excel 2016 were used to assist with the graphing (Figure 1).

## 3. Results and Discussion

### 3.1. Characteristics of the Publications

**Figure 2:**
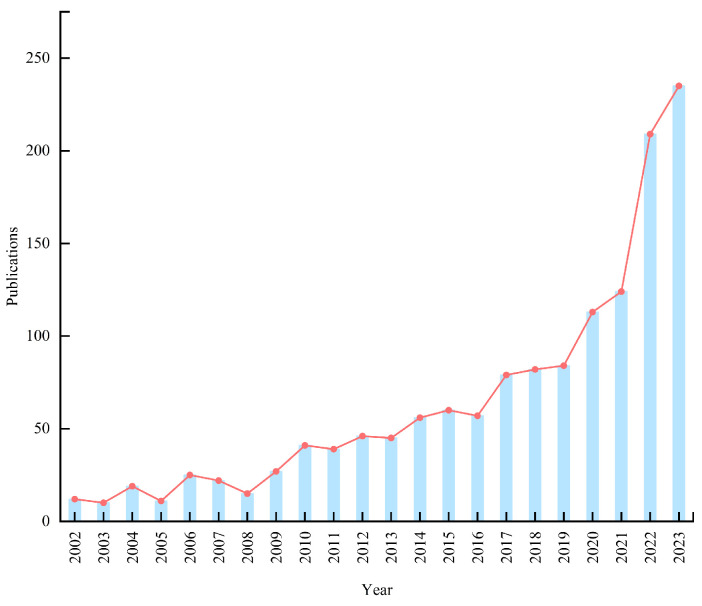
Distribution of publications on carbon sources and sinks of farmland ecosystems published from 2002 to 2023.

Figure 2 shows the annual publication volume and publication trends in research on carbon sources and sinks in farmland ecosystems from 2002 to 2023. Only 99 studies (99; 7%) were published before 2008. Afterward, the number of publications increased, potentially related to the start of the Bali Road Map negotiations in 2007. The negotiations of the “Bali Road Map” incorporated all developing countries into the emission reduction framework and proposed a mechanism of “measurable, reportable, and verifiable” (MRV) actions to drive carbon emission reductions. This directly heightened global attention to the dynamics of carbon sources and sinks, promoting research in this field. Thereby, the adoption of the Paris Agreement in 2015, which clarified countries’ emission reduction commitments, provided a strong push for research in this field. By 2019, more than half of the publications (730; 51.74%) had been published. The period from 2021 to 2023 is the most productive (carbon neutrality has been proposed globally), with over 40% of the studies (568; 40.2%) published in this period (Figure 2).

### 3.2. Publication Output Analysis

From 2002 to 2023, 309 journals published research articles on carbon sources and sinks in farmland ecosystems (data from the WOS platform). *Science of the Total Environment* (93 publications; 2800 citations), *Agriculture Ecosystems & Environment* (74 publications; 4903 citations) and *Global Change Biology* (93 publications; 8270 citations) were the journals with the highest numbers of publications and citation rates (Table 1). *Global Change Biology* had 1.69 times more citations than *Agriculture Ecosystems & Environment* and 2.9 times more citations than *Science of the Total Environment*. This highlights these journals’ strong international academic influence in the field of carbon sources and sinks in farmland ecosystems, particularly that of *Global Change Biology*, which stands out as an important platform for academic exchange. Additionally, the studies in these journals primarily focus on the environmental sciences, ecosystems, and climate change, reflecting the increased attention to carbon sources and sinks in farmland ecosystems in terms of their ecological and environmental impacts in the context of global climate change.

### 3.3. Analysis by Author, Institution, Country (Region), and Subject Area 

**Figure 3:**
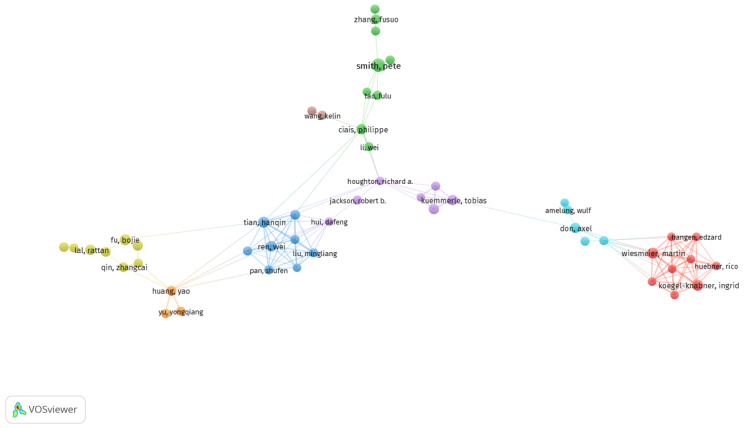
Authors’ co-authorship network analysis. Each cluster represents the network of Authors working together.

In this study, we employed VOSviewer software for author cluster analysis to identify the most representative scholars in the field of carbon sources and sinks in farmland ecosystems (Table 2) and their collaborations (Figure 3). We set a minimum of five publications per author, with 73 out of 6549 authors meeting the criteria. For each of the 73 authors, the total link strength of co-authorship with others was calculated, and those with the highest link strength were selected. The authors were clustered into eight categories, each marked with a different color in Figure 2. Although Professor Pete Smith (20 publications) and Professor Fu Bojie (11 publications) are the top contributors in terms of the number of publications, they have fewer collaborations (Figure 3). On the other hand, Tian Hanqin, Ingrid Kögel-Knabner, and Martin Wiesmeier both have a notable number of publications and have collaborated extensively with other researchers.

**Figure 4:**
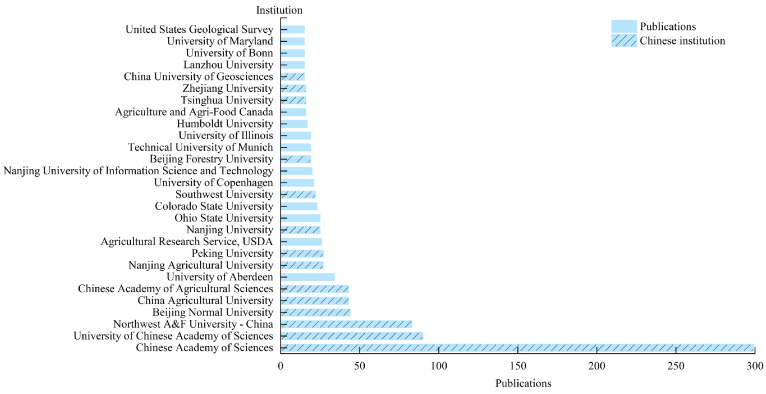
Research institutions or organizations with 15 or more publications.

Publishing organizations are the driving force behind academic research and can provide insight into institutions’ importance in a particular field. A total of 1673 organizations worldwide have published studies on carbon sources and sinks in farmland ecosystems, reflecting the global interest in research on the carbon cycle in farmland ecosystems. Over the last two decades, 27 organizations have published more than 15 publications each (Figure 4), with the Chinese Academy of Science (402 publications; 28.49%) having the highest output, followed by the Northwest A&F University (83 publications; 6%), Beijing Normal University (44 publications; 3%), China Agricultural University (43 publications; 3%), and the Chinese Academy of Agricultural Sciences (43 publications; 3%). It is noteworthy that 15 of the 27 institutions are from China, reflecting the nation’s commitment to reducing carbon and emissions in farmland ecosystems and indicating the vital role played by Chinese research institutions in this field.

**Figure 5:**
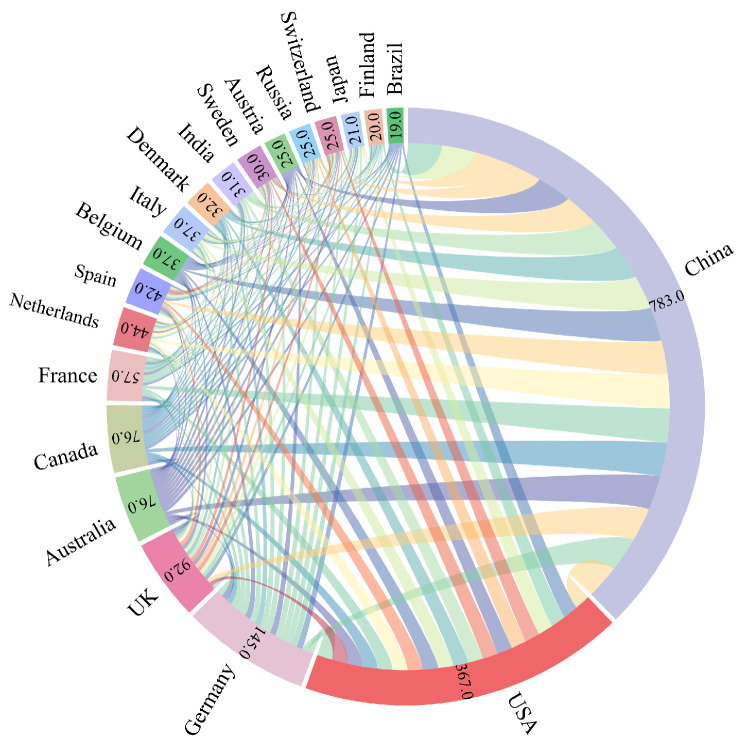
Top 20 countries by volume of collaborative research. The lines of different colours represent cooperation between different countries.

Collaboration in scientific research enhances the capacity for disciplinary integration, increases the impact of research, and promotes deeper theoretical mechanisms [12]. Publications co-authored by Chinese scholars in collaboration with researchers from other countries represent 39.08% of China’s total publication output, underscoring China’s extensive engagement in international cooperative efforts in this field (Figure 5). In addition, the United States, Germany, Canada, and Australia are also countries that cooperate closely with other countries in scientific research (Figure 5). Most of the countries with a high number of publications featuring international collaborations are major agricultural countries with advanced scientific and technological capabilities, and all of them are parties to the Paris Agreement, which indicates that they are committed to reducing carbon emissions [18]. This suggests that the international environment, politics, and the strength of scientific research have shaped the evolution of this research arena.

**Figure 6:**
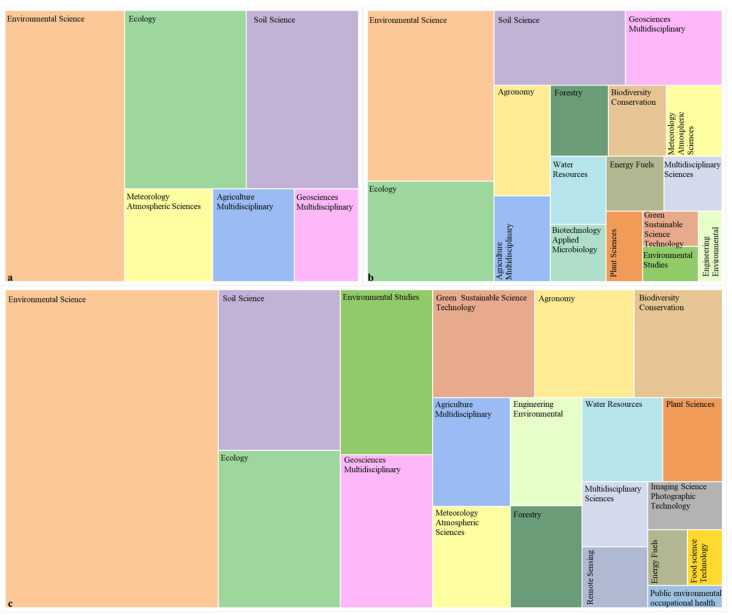
Disciplines with over ten publications on carbon sources and sinks of global farmland ecosystems: (**a**) 2002–2009; (**b**) 2010–2016; (**c**) 2017–2023. Different colours represent different disciplines.

Interdisciplinarity has emerged as a significant approach in modern scientific research [19]. Engaging in interdisciplinary exchanges and collaborations not only advances systematic theoretical research but also propels the rapid development of the field [20]. In the subject matter of farmland ecosystems, the study of carbon sources and sinks is primarily dominated by the disciplines of environmental science, ecology, and soil science, with environmental science being markedly predominant (Figure 6). This dominance reflects the role of farmland ecosystems in responding to global climate change. Ecology and soil science have always been essential disciplines, underscoring the ongoing attention to ecosystem services, biodiversity conservation, and soil carbon sequestration. Over time, the field of water resources has highlighted the study of the interplay between climate change and agricultural soil hydrological processes, attracting growing attention. Moreover, the integration of biotechnology, microbiology, botany, and remote sensing has provided new research perspectives and technical approaches for understanding the carbon sequestration mechanisms within farmland ecosystems.

### 3.4. Research Theme Analysis

**Figure 7:**
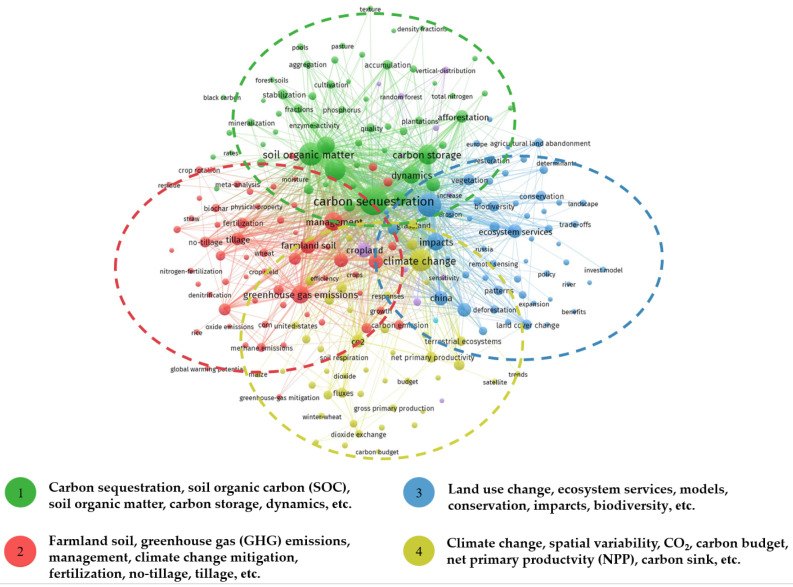
Co-occurrence map of keyword clusters. Four major themes in the literature are based on research topics defined by all the keywords. The circle size represents the occurrence of keywords, and high occurrences usually have high link strengths.

Keywords are a brief summary of the core content of studies, capable of reflecting the main research content in the field of study [21]. This study employed the co-word analysis functionality of VOSviewer to identify the primary research directions within the field. With a minimum of 10 set as thresholds, 228 out of 5752 keywords were selected (Figure 7). In this analysis, the circle size represents keyword frequency, and the line thickness indicates the strength of keyword associations. Keyword clustering separates the current research topic areas of carbon sources and sinks in farmland ecosystems into four categories, and four overarching themes, marked in different colors, become apparent (Figure 7).

#### 3.4.1. Study on Soil Organic Carbon Sequestration Mechanisms

Theme 1 focuses on carbon sequestration, SOC, carbon storage, soil organic matter (SOM), and their dynamics (Figure 7). Based on the total link strength of node connections, this research cluster is primarily focused on SOC dynamics, the mechanisms of SOC sequestration, and the stocks of SOC. Increasing the SOC content in agricultural fields has a dual effect: it enhances soil productivity and mitigates the rise in atmospheric CO_2_ concentration [5]. The dynamic processes of SOC input, decomposition, migration, transformation, and accumulation in soils are influenced by various factors, including natural conditions, human activities, and time. For example, earthworm bioturbation affects soil carbon storage, particularly near earthworm burrows, although their activity does not significantly increase subsoil carbon [22]. However, it facilitates the transport of fresh organic matter to deeper soil layers [22]. When natural ecosystems are converted into farmland, the rate of SOC loss varies depending on the type of cropping system established, with perennial woody plants resulting in lower organic carbon loss compared to annual crops [23]. The commonly used methods for studying SOC dynamics include isotope labeling and modeling approaches [24,25]. Isotope labeling provides direct measurement and tracking of organic carbon dynamics, but it is costly [26]. Modeling integrates a large number of climate and soil data to simulate crop growth and the turnover of SOC, enabling large-scale predictions and analyses. Nevertheless, the accuracy of this approach is influenced by the parameters and quantity of input data [27]. Over the years, scholars have extensively advanced in understanding the mechanisms of SOC sequestration. Six et al. systematically summarized the main factors influencing the dynamics of soil aggregates and organic matter [28]. They proposed a conceptual model of the “life cycle” of a macroaggregate, highlighting the formation of new microaggregates and the interplay between organic carbon accumulation and mineralization. This process involves the cyclical process of aggregate formation, turnover, and disruption. In the formation phase, fresh residues enter the soil, and intra-aggregate particulate organic matter (iPOM) is incorporated into macroaggregates. During turnover, iPOM decomposed into finer particles, which are then bound by clay and microbial products, forming more stable microaggregates. Disruption occurs when the degradation of microbial products and other binding substances leads to a reduction in the stability of macroaggregates, causing their breakdown, releasing microaggregates, and initiating a new cycle of macroaggregate formation [29]. This cycle shows that the turnover of aggregates is closely related to the dynamic changes in particulate organic matter [28,29]. Christensen challenged the traditional size-based classification of aggregates, proposing a theory of structural division with three levels: Level 1 structure—soil organic–mineral complexes; Level 2 structure—secondary complexes formed by the reaggregation of primary organomineral complexes; and Level 3 structure—intact soil in situ [30]. This theory provides a multi-scale perspective for a deeper understanding of organic matter turnover and storage in soil. Previous studies have suggested that plant-derived carbon is the primary initial source for the formation of SOM, which undergoes microbial decomposition reactions and is subsequently transformed into stable SOC [31]. Advances in high-throughput sequencing and omics technologies have revealed a crucial role of soil microorganisms in the formation and stabilization of SOC. Liang et al. proposed the “microbial carbon pump” (MCP) theory, suggesting that microbial-derived carbon is an essential source of SOC pools alongside plant-derived residues. The theory primarily consists of three aspects: First, soil microorganisms decompose or transform macromolecular plant organic residues through the ex vivo modification pathway and transport them into the soil. Second, soil microorganisms convert small molecular substrates into their biomass through in vivo turnover, with the process of microbial growth, reproduction, and death cycling and assimilation, thereby delivering microbial-derived organic carbon into the soil. Third, microbial communities continuously produce microbial-derived carbon through the iterative process of growth and death, which is driven by the “entombing effect” [32]. The proposal of this theory clarifies the regulatory mechanisms by which soil microorganisms influence soil carbon sequestration, thereby providing a new perspective for research on carbon cycling in farmland ecosystems [32]. Xiao et al. proposed the theory of the “soil mineral carbon pump” (MnCP), which suggests that soil minerals can interact with SOM through various abiotic processes such as adsorption, occlusion, aggregation, redox reactions, and polymerization. Adsorption refers to SOM adhering to mineral surfaces, while occlusion utilizes SOM being trapped within mineral crystals. In addition, aggregation occurs when minerals and organic matter bind to form aggregates. Redox reactions involve SOM reacting with minerals via direct or indirect electron transfer, resulting forming radicalized organic compounds that promote polymerization. Finally, polymerization is catalyzed by metal oxides or clay minerals, converting the reducing sugars or free amino acids into complex aromatic hydrocarbon macromolecules [33]. These interactions form organo-mineral complexes that lower the bioavailability and degradation rate of SOM, thus converting labile SOC derived from plants or microorganisms into more stable forms. This theory enhances our understanding of the abiotic mechanisms of soil carbon sequestration [33]. The MCP and the MnCP exhibit a “sequential” temporal relationship, where the microbial process of plant-derived carbon transformation and organic carbon production is considered the “upstream” processes of SOC formation. Soil minerals play a stabilizing role in the SOC formed through MCP, representing the “downstream” process. However, soil minerals can also stabilize SOC that has not undergone MCP transformation. Therefore, both the MCP and MnCP are interconnected, and each has unique characteristics. Regardless of how the “dual pumps” interact, each contribution to SOC sequestration will be influenced by both natural factors and human activities [34,35]. Future research on carbon cycling in farmland ecosystems should prioritize examining the synergistic and interactive effects of the “dual pumps” in the transformation and sequestration of SOC. Furthermore, assessing SOC stock change across global, national, and regional scales is vital for evaluating the potential of farmland ecosystems in mitigating climate change [34]. Scholars have estimated SOC stocks at various scales, including profile, plot, regional, and global scales, through long-term field experiments, literature reviews, and modeling approaches (both empirical and mechanistic). These efforts have helped quantify the global and regional SOC stocks and their carbon sequestration capacity [6].

#### 3.4.2. Research on Greenhouse Gas Monitoring and Estimation

Theme 2 focuses on farmland management, GHG emissions, ecosystems, and climate change mitigation (Figure 7). The total link strength of node connections indicates that the majority of studies revolve around the GHG emissions and carbon sequestration effects of different agricultural management practices. The Kyoto Protocol recognized agricultural land management as one of the key measures for reducing GHG emissions during the first commitment period, advocating the adoption of sustainable farming practices to enhance soil carbon sequestration [35,36]. This has garnered widespread attention, with scholars conducting extensive research to verify that agricultural management practices enhance soil carbon sequestration capacity and effectively reduce GHG emissions. Among these practices are fertilization methods (organic fertilizer applications, precision fertilization) [37], tillage practices (no-tillage, conservation tillage) [38], optimizing crop rotation schemes [39], returning crop residues to the soil, and so on [40,41]. By 2011, conservation tillage practices had been implemented on 1.25 billion hectares of farmland worldwide, excluding Antarctica, with widespread adoption across all continents [42,43,44,45]. Countries such as Brazil, Argentina, and Paraguay in South America have actively promoted conservation tillage through policy initiatives [46]. By the end of the 20th century, the adoption of conservation tillage practices and the Conservation Reserve Program (CRP) in the United States transformed agricultural land from a carbon source into a carbon sink [47,48]. In a long-term field experiment, Tian et al. found that conservation tillage does not increase GHG emissions in the context of global warming. Instead, it enhances microbial growth efficiency, promotes the accumulation of fungal necromass carbon, and facilitates the formation and sequestration of SOC [49]. This confirms that conservation tillage is a widely recognized and effective measure for carbon sequestration and GHG reduction globally. However, compared to tillage practices, the promotion costs and technical challenges associated with agricultural management measures such as fertilization management and cropping systems are relatively high. Consequently, there is still no consensus on the effectiveness of these measures in reducing GHG emissions and enhancing carbon sequestration, necessitating further research and exploration. Scholars have developed measurement methods that can be broadly categorized into two types to evaluate GHG emissions under different agricultural management practices: direct and indirect methods. Direct methods primarily include the chamber method and gas chromatography (GC) [50], while indirect methods mainly consist of the micrometeorological method and the mass balance method [51]. The chamber method is widely used for measuring soil CO_2_, CH_4_, and N_2_O emissions [14]. For example, Chi et al. used this method to examine the effects of no-till and conventional tillage cropping systems on carbon and water fluxes in the inland Pacific Northwest of the United States [52]. The findings revealed that no-till farming could reduce CO_2_ emissions and contribute to the sustainable development of the farmland ecosystem. Compared to the chamber method, the micrometeorological method is an open-ended and effective method for studying the material transport and energy exchange between vegetation and the atmosphere [53]. Additionally, this method plays a crucial role in analyzing ecological processes and determining parameters for remote sensing models [54]. The most commonly used micrometeorological technique is the vorticity correlation method, which calculates gas exchange flux within the system by measuring turbulence parameters [14]. For example, Xiao et al. utilized eddy covariance flux and meteorological data to study annual carbon fluxes in the Heihe River Basin of China [55]. The results demonstrated that annual carbon fluxes in forest and farmland plots were higher than those in grassland plots, while fluxes in coastal wetland plots were comparable to or slightly exceeded those in forest plots. Furthermore, assessing GHG emissions across farmland management practices and regions using different carbon emission accounting methods has become a crucial approach for quantifying GHG emissions. The primary GHG accounting methods currently include direct measurement, emission factor, mass balance, life cycle, and modeling approaches (e.g., farmland ecosystem carbon cycle model). Each method has distinct advantages and limitations (Table 3), and the selection of an appropriate method should align with the research objectives.

#### 3.4.3. Research on Ecosystem Services

Theme 3 concentrates on land use change, ecosystem services, conservation, and models (Figure 7). The total link strength of node connections indicates that most studies focus on the loss of ecosystem services caused by land use changes and the internal relationships within farmland ecosystem services. Land use and cover changes can significantly alter the energy balance and material cycling processes of ecosystems, leading to changes in ecosystem functions and affecting the supply of ecosystem services [56]. Urban expansion has encroached upon a large amount of farmland, resulting in a series of ecological issues such as reduced food production, carbon sequestration, and biodiversity loss [57]. The scarcity of farmland ecosystem services has become increasingly prominent, and their multifunctionality has garnered attention, leading to widespread interest in the study of these services and their valuation [58]. Farmland ecosystems provide various services, but people often aim to maximize the utilization of a single ecosystem service [56]. However, when multiple ecosystem services are utilized competitively, it typically leads to trade-offs within the ecosystem services themselves [59]. For example, crop yield increases often come at the cost of other ecosystem services [60]. However, sustainable human development requires the synergistic advancement of multiple ecosystem services [61]. As a result, achieving coordinated development across these services and developing scientific trade-off decision-making methods have become central research directions. Moreover, systematic evaluation methods have been developed to quantify and assess the dynamics of farmland ecosystem services under different management practices. These methods mainly include participatory approaches, sample plot observation, empirical models, mechanistic models, and value assessment methods. Each method focuses on the evaluation of different farmland ecosystem service functions based on their distinct principles or approaches [60]. In addition, scholars have proposed a range of assessment methods to quantify the dynamics of these services and evaluate their change under different management and agricultural practices. The sample plot observation method is commonly used to evaluate farmland ecosystem services such as crop pollination and pest control [62]. The Cycle model focuses on the water, carbon, and nitrogen balance of soil-crop systems under climate and farmland management influences. It is primarily used to evaluate farmland ecosystem service functions related to food production (provisioning services) and climate change mitigation (regulating services) [63].

#### 3.4.4. Spatiotemporal Evolution and Driving Factors of Carbon Sources and Sinks in Farmland Ecosystems

Theme 4 focuses on research related to climate change, carbon sinks, primary productivity, CO_2_, and spatial variability (Figure 7). According to the total link strength of node connections and clustering results, this theme centers on exploring the spatiotemporal heterogeneity of carbon stocks and carbon emissions in farmland ecosystems, alongside their drivers in the context of climate change. The carbon pools in farmland ecosystems mainly consist of the soil carbon pool (measured by SOC) and the crop carbon pool (assessed by NPP and crop biomass) [64]. The primary GHG emission sources in these ecosystems include soil respiration, methane (CH_4_) emissions from paddy fields, nitrous oxide (N_2_O) emissions from agricultural soils, and GHG emissions resulting from agricultural inputs and farm practices [65,66,67,68]. The carbon cycle in farmland ecosystems is a complex process influenced by factors such as climate, topography, crop type, soil properties, and anthropogenic activities [5,69]. Variations in these factors can modify carbon exchanges among different carbon pools within the ecosystem, leading to “carbon source” or “carbon sink” effects [70]. Moreover, numerous studies have demonstrated that the factors controlling carbon sources and sinks in farmland ecosystems exhibit varying influences across spatiotemporal scales, highlighting the scale effect in these control factors [71,72,73]. At the microscale, soil properties and farming management practices are key drivers of GHG emissions and SOC sequestration [74], as shown by numerous laboratory and field experiments [2,73]. At the mesoscale, such as in basins and geographical regions, climate, topography, and anthropogenic activities primarily influence GHG emissions and SOC sequestration [73]. For example, Smith et al. demonstrated that changes in farmland management were the primary drivers of declining SOC levels in farmland across England and Wales during the late 20th century [75]. The impacts of climate change and anthropogenic activities are more pronounced at the macroscale, such as global and continental [73,76]. The research conducted by Zhou et al. indicated that temperature is leading factor driving the spatiotemporal variability in future topsoil SOC storage [77]. Furthermore, high-latitude regions, which are more warming rapidly experience the most pronounced effects on topsoil SOC loss [77,78,79]. According to Wang et al., the efficiency of nitrogen fertilizer use is the primary contributor to global N_2_O emissions [80]. However, climate change, particularly rising temperatures, accelerates soil microbial metabolic activity, enhancing soil organic nitrogen decomposition and increasing N_2_O emissions [81]. Through a meta-analysis, Yu et al. found that non-continuous flooding significantly reduces GHG emissions from paddy fields worldwide [81]. Research on carbon sequestration in farmland crops remains underdeveloped due to ongoing controversy surrounding the carbon pools in these systems [11,82]. There is a need for further investigation into the spatiotemporal variation patterns and driving factors of crop carbon pools. Additionally, there is currently no consensus on how different factors influence carbon sources and sinks at various scales within farmland ecosystems. Many studies tend to focus on the impact of specific categories or individual factors rather than the broader spatiotemporal heterogeneity of these processes.

### 3.5. Research Hotspot Evolution and Trend Analysis

#### 3.5.1. Research Hotspot Evolution Analysis

**Figure 8:**
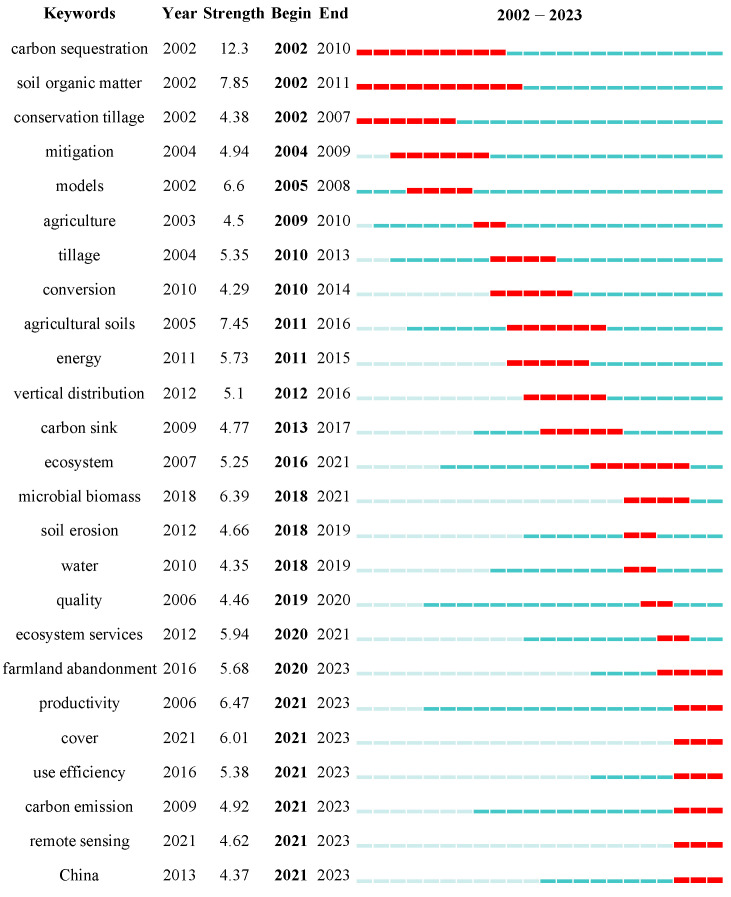
Evolution of the top 25 keywords during 2002–2023. Strength is a measure of the degree of a burst event. The larger the value, the more significant the keyword is in the research field. The red line represents the year with active burst words, and the cyan-blue line represents the year with inactive burst words.

Burst words refer to terms that suddenly become hotspots or are more frequent during a specific period, signifying the research hotspots at different stages within a particular field [83,84]. Using the CiteSpaces burst analysis function, the top 25 burst keywords in the study of carbon sources and sinks in farmland ecosystems were identified (Figure 8). Key terms mainly include carbon sequestration, SOM, models, cropland, microbial biomass, productivity, and cover, each with a burst strength exceeding 6.0, highlighting focal areas in this research field. Carbon sequestration and SOM exhibited the most extended and intense burst durations, indicating a significant focus on SOC sequestration in farmland soils, which aligns with the findings of the thematic analysis. Similarly, models showed high burst intensity during the same period, reflecting the prominence of modeling research as a mainstream methodological approach in this field. Additionally, tillage practices, including conservation and conventional tillage, demonstrated strong burst strength, highlighting increasing attention to farmland management practices and their potential for carbon reduction and sequestration. Since 2018, microbial biomass, ecosystem services, and carbon emissions have emerged as prominent frontier research areas. After the proposed concept of “carbon neutrality” was introduced globally, China became a hotspot for research, indicating the high importance the Chinese government and scholars place on the carbon neutrality goal. Furthermore, by reviewing the literature, we found that the number of publications from China surged after 2018. Between 2018 and 2023, Chinese scholars contributed 25.02% of the papers in this field (353 articles). The frequent occurrence of “China” as a research label for case study regions also substantiates China’s strong commitment to carbon sequestration and emission reduction. In addition, remote sensing technology emerged in this field in 2021 and quickly gained attention, enabling the acquisition of multi-scale data on carbon fluxes. This advancement has promoted research on the spatiotemporal variations of carbon sequestration in farmland ecosystems.

#### 3.5.2. Research Fronts and Research Trends

**Figure 9:**
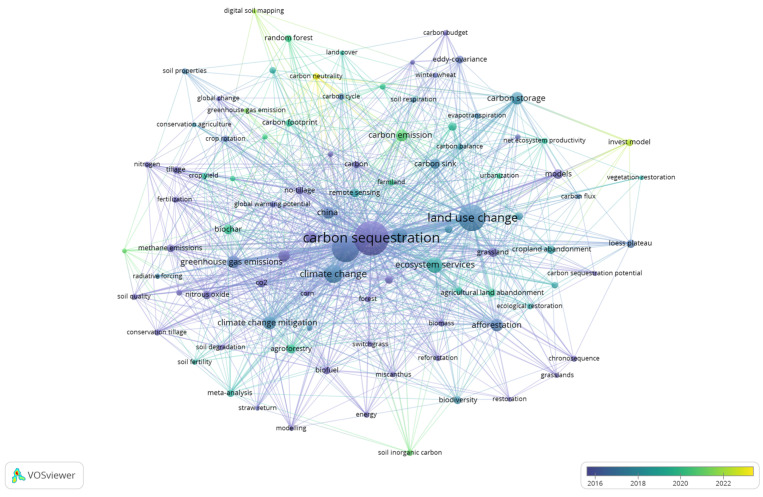
Temporal evolution trend of author keywords in carbon sources and sinks research of farmland ecosystem. The size of the circles represents the frequency of keyword occurrences, different colors represent different years, and lines indicate the link strength between keywords.

Author keywords, specified by authors during article publication, directly convey their understanding and definition of the research topic, effectively highlighting the key focus areas and emerging trends within a specific research field. Therefore, this paper explores the current research frontiers and trends through the temporal map of author keyword co-occurrence using VOSviewer. Setting the minimum occurrence frequency of keywords to 8 and the minimum link strength to 8, of all the 3633 author keywords, 95 met the threshold. The top 10 author keywords were carbon sequestration (N = 340), SOC (N = 217), land use change (N = 205), climate change (N = 100), cropland (N = 80), GHG emissions (N = 61), climate change mitigation (N = 52), ecosystem services (N = 69), and agriculture (N = 37) (Figure 9). The total link strengths of “carbon sequestration” and “SOC” were 536 and 397, respectively, far surpassing other keywords and indicating they are the core keywords, highly correlated with other keywords. The temporal evolution of keywords showed that research on carbon sources and sinks in farmland ecosystems has changed over time. Initially, studies primarily focused on the carbon sequestration potential and effects of farmland soils, while later research shifted attention to soil organic carbon loss resulting from land use changes. Recent studies mainly emphasize reducing carbon emissions and carbon footprints to achieve agricultural carbon neutrality. Biochar has gained significant attention as an essential carbon sequestration measure for its potential in carbon sequestration due to its ability to store carbon and reduce emissions. Farmland ecosystem services, closely linked to sustainable human development, are a critical area of research. The Invest model is an essential tool for assessing the value of ecosystem services.

## 4. Future Research Directions

Farmland ecosystems are crucial in climate change mitigation, enhancing carbon sequestration, and reducing GHG emissions to achieve carbon neutrality. Based on the current challenge in carbon sources and sinks of farmland ecosystem research, we propose the following focuses for future research.

**Further in-depth exploration of soil carbon sequestration mechanisms in farmland ecosystems is needed.** Various factors, including crops, microorganisms, soil fauna, agricultural management practices, and parent material, all influence soil carbon sequestration in the farmland ecosystem. While the role of soil minerals and microorganisms in carbon sequestration is understood, research on the dominant roles and interactions of the MCP and MnCP in SOC storage under different climate types and agricultural practices in the context of global warming is still lacking. Furthermore, although it is well established that microorganisms regulate the chemical composition of SOC through processes such as “ex vivo modification” and “in vivo turnover” and control the dynamic storage of soil-stable organic carbon via “priming effects” and “entombment effects”, soil fauna also influence the formation and accumulation of microbially derived organic carbon. However, there is currently a lack of understanding and assessment regarding the impact of soil fauna on the entire process chain of microbially derived carbon sequestration.

**The focus should be on studying the interactive effects of drivers on the spatiotemporal changes in carbon sources and sinks within farmland ecosystems**. Due to variations in management practices and natural geographic environments, the regulatory mechanisms and cycling patterns of carbon sources and sinks differ significantly across regions. Most studies on the spatiotemporal variation in farmland SOC pools focus on specific factors influencing carbon sources and sinks. However, the carbon cycle in farmland ecosystems involves multiple factors, such as temperature, precipitation, topography, soil respiration, and farming management practices. These factors interact in intricate ways, collectively influencing carbon sequestration and emissions in farmland ecosystems. There is a notable lack of research on the spatiotemporal variation in crop carbon pools in farmland ecosystems. Some scholars argue that crops have minimal carbon sink effect due to the short growing period of crops and the release of CO_2_ back into the atmosphere after harvest [85,86]. Consequently, crops are often assumed to contribute zero carbon sequestration when estimating the carbon stock of farmland ecosystems [6,87]. However, some scholars have proposed that crops play an important role in carbon sequestration within the system before harvest. Furthermore, after crops are harvested, agricultural activities continue, and the corresponding carbon is transferred from the atmosphere to the soil and other components of the system [11,68]. It is worth noting that recent research has shown that the pathways and forms of crop carbon sequestration have shifted in the context of global change. Firstly, the construction of high-standard farmland has improved the productivity of farmland in China [88]. Secondly, organic carbon from crop residues and straw decomposition is accelerated into the soil, increasing SOC accumulation and forming a relatively stable carbon sink [89]. Thirdly, the widespread use of agricultural fertilizers has significantly increased crop growth rates, boosted food production, and accelerated the sequestration of CO_2_ from the atmosphere into the farm ecosystem [90]. In conclusion, the farmland crop carbon pool can be considered a tangible carbon reservoir with significant carbon absorption functions, like the vegetation carbon pools in other ecosystems. Therefore, future research should focus on the combined effects of multiple factors, such as temperature rise, changes in precipitation, shifts in agricultural management practices, and topography, at global and regional scales to comprehensively understand the mechanisms behind the spatiotemporal changes and heterogeneity of carbon sources and sinks in farmland ecosystems under climate change.

**Improving carbon accounting methods for farmland ecosystems.** Different methods used for carbon accounting at the regional scale lead to significant uncertainties, making it challenging to compare results across various approaches. It is recommended to assess the weakness and strength of different methods. A “multi-data, multi-process, multi-scale, multi-method” integration should be adopted to establish a “sky-space-ground” system for measuring carbon sources and sinks in farmland ecosystems to improve estimation approaches. This includes strengthening remote sensing observations (vegetation data), developing a comprehensive network to monitor atmospheric CO_2_ concentrations, and enhancing soil carbon pool monitoring and inventory, thus providing a solid data foundation for farmland ecosystems.

**Emphasize research on the internal relationships and value assessment of farmland ecosystem services.** In the context of global warming and the escalating food security crisis, adjusting farmland land use intensity, optimizing spatial land layout, and promoting the coordinated development of provisioning, regulating, and supporting services in the farmland ecosystem are vital for mitigating climate change, ensuring food security, preserving biodiversity, and supporting regional sustainable development.

## 5. Conclusions

In the context of climate change, research on carbon sources and sinks in farmland ecosystems has become one of the most pressing global environmental issues. This study employs bibliometric methods using CiteSpace and VOSviewer, based on the Web of Science database, to conduct a bibliometric analysis and systematic review of farmland ecosystem carbon source and sink research. This study also offers a reproducible methodological framework for constructing knowledge maps of complex ecological issues by providing a data-driven foundation for defining research directions and development pathways. The main research findings are presented below.

Over the past 22 years, research on farmland ecosystem carbon sources and sinks has shown continuous growth, with a notable increase in the last three years. China, the United States, and Germany have made significant contributions and frequently engaged in international collaborations with representative scholars, including Ingrid Kögel-Knabner, Martin Wiesmeier, and Hanqin Tian. This field has developed into a multidisciplinary research domain, with *Global Change Biology* and *Agriculture, Ecosystems & Environment* emerging as key academic exchange platforms.

The research primarily focuses on soil organic carbon (SOC) sequestration mechanisms and GHG measurement methodologies, leading to establishing differentiated assessment systems that confirm the significant carbon sequestration and emission reduction benefits of conservation tillage. However, climate change, land use transformation, and variations in farmland management practices result in pronounced spatiotemporal heterogeneity in carbon source–sink effects. Among these factors, climate change is the primary determinant of large-scale carbon balance, while farmland management practices play a crucial regulatory role in multi-scale effects.

Nevertheless, current research still has limitations. The dominant roles and interactive mechanisms of soil aggregates, mineral-associated carbon, and microbial metabolic products under different regional conditions remain unclear. Disparities in models and methodologies contribute to high uncertainty in assessments, and the interactive mechanisms among driving factors are yet to be fully elucidated. Future research should deepen investigations into soil organic carbon stabilization mechanisms, refine carbon source–sink accounting methodologies, and enhance the analysis of interactions among driving factors. These efforts will provide theoretical support for sustainable agricultural development and achieving “dual carbon” goals.

## Figures and Tables

**Table 1:** Top 10 journals with published articles in the field of carbon sources and sinks in farmland ecosystems.

Ranking	Journal	Number of Publications	Proportion of Publications (%)	Total Citations
1	*Science of the Total Environment*	93	6.59	2800
2	*Agriculture Ecosystems & Environment*	74	5.24	4903
3	*Global Change Biology*	56	3.97	8270
4	*Catena*	43	3.05	1203
5	*Journal of Cleaner Production*	40	2.83	939
6	*Ecological Indicators*	39	2.76	1375
7	*Land*	35	2.48	168
8	*Sustainability*	34	2.41	360
9	*Geoderma*	31	2.20	1434
10	*Land Degradation & Development*	30	2.13	1197

**Table 2:** Top 10 authors in the field of carbon sources and sinks of farmland ecosystems.

Author	Number of Publications	Total Citations in the Literature	Average Year of Publication
Smith, Pete	20	2824	2016
Tian, hanqin	12	870	2014
Fu, bojie	11	1473	2013
Koegel-knabner, Ingrid	11	821	2016
Wiesmeier, Martin	11	828	2017
Ciais, Philippe	10	582	2020
Deng, Lei	10	1054	2016
Don, Axel	10	916	2018
Kuemmerle, Tobias	10	1054	2015
Prishchepov, alexander V.	10	643	2017

**Table 3:** Different GHG emission accounting methods [12,13,14,51,52,55].

Method	Advantages	Shortcomings	Scale	Applications
Measurement method	① Easy to operate; ② results are precise and reliable.	① Data acquisition challenges; ② expensive human and material resources; ③ vulnerability.	Microscopic	Microscopic or simple ecosystem.
Emission factor method	① Easily operated and understood; ② established accounting formulas, activity data, and emission factor databases are available; ③ straightforward data collection.	Lack of capacity for emission system changes.	Macroscopic; mesoscopic; microscopic	Socioeconomic emission sources; typically complex or simplified natural emission sources or carbon sinks.
Mass balance method	Clearly distinguishes between emission sources.	① Cumbersome intermediate procedures for emissions to be taken into account; ② prone to systematic errors; ③ obtaining data is difficult.	Macroscopic; mesoscopic	Emission equipment is frequently updated and highly precise; natural emission sources are complex.
Life cycle method	Assesses the total GHG emissions arising from all activities and inputs across the full life cycle of a production or consumption process.	Cumbersome process.	Mesoscopic; microscopic	Features a relatively complete and systematic production process.
Modeling method	① Quantitatively distinguishes the contribution of different factors to changes in carbon sources and sinks; ② predicts future changes in carbon sources and sinks; ③ reflects the material cycling processes of ecosystems.	① Complex model structure with difficult parameter adjustments; ② limited or simplified consideration of ecosystem management’s impact on carbon cycling processes; ③ many models neglect lateral carbon transfer processes, such as watershed transport.	Macroscopic; mesoscopic; microscopic	Large regions, various administrative divisions, field scales, and multi-scale ecosystems.

## Data Availability

Publications on carbon sources and sinks in farmland ecosystems referenced in this work were sourced from Web of Science (https://webofscience.clarivate.cn/wos/woscc/advanced-search), accessed on 19 February 2024.

## Abbreviations

The following abbreviations are used in this manuscript:

SOC	Soil organic carbon
GHG	Greenhouse gas
GIS	Geographical Information System
NPP	Net primary productivity
WOS	Web of Science
WOSCC	Web of Science Core Collection
SSCI	Social Sciences Citation Index
SCI-EXPANDED	Science Citation Index Expanded
SOM	Soil organic matter
iPOM	Intra-aggregate particulate organic matter
MCP	Microbial carbon pump
MnCP	Soil mineral carbon pump
CRP	Conservation Reserve Program
GC	Gas chromatography

## References

[1] Fang J., Yu G., Liu L., Hu S., Chapin F.S. (2018). Climate Change, Human Impacts, and Carbon Sequestration in China. Proc. Natl. Acad. Sci. USA.

[2] Della Chiesa T., Northrup D., Miguez F.E., Archontoulis S.V., Baum M.E., Venterea R.T., Emmett B.D., Malone R.W., Iqbal J., Necpalova M. (2024). Reducing Greenhouse Gas Emissions from North American Soybean Production. Nat. Sustain..

[3] Jobbágy E.G., Jackson R.B. (2000). The Vertical Distribution of Soil Organic Carbon and Its Relation to Climate and Vegetation. Ecol. Appl..

[4] Intergovernmental Panel on Climate Change (IPCC) (2022). Land–Climate Interactions. Climate Change and Land: IPCC Special Report on Climate Change, Desertification, Land Degradation, Sustainable Land Management, Food Security, and Greenhouse Gas Fluxes in Terrestrial Ecosystems.

[5] Lal R. (2004). Soil Carbon Sequestration Impacts on Global Climate Change and Food Security. Science.

[6] Yang Y.H., Shi Y., Sun W.J., Chang J.F., Zhu J.X., Chen L.Y., Wang X., Guo Y.P., Zhang H.T., Yu L.F. (2022). Trestrial carbon sinks in China and around the world and their contribution to carbon neutrality. Sci. Sin..

[7] Clune S., Crossin E., Verghese K. (2017). Systematic Review of Greenhouse Gas Emissions for Different Fresh Food Categories. J. Clean. Prod..

[8] Lal R. (2003). Global Potential of Soil Carbon Sequestration to Mitigate the Greenhouse Effect. Crit. Rev. Plant Sci..

[9] West T.O., Marland G. (2002). Net Carbon Flux from Agricultural Ecosystems: Methodology for Full Carbon Cycle Analyses. Environ. Pollut..

[10] Jiang Z., Zhong Y., Yang J., Wu Y., Li H., Zheng L. (2019). Effect of Nitrogen Fertilizer Rates on Carbon Footprint and Ecosystem Service of Carbon Sequestration in Rice Production. Sci. Total Environ..

[11] Wei H., Wu L., Chen D., Yang D., Du J., Xu Y., Jia J. (2024). Rapid Climate Changes Responsible for Increased Net Global Cropland Carbon Sink during the Last 40 Years. Ecol. Indic..

[12] Li H., Jin X., Zhao R., Han B., Zhou Y., Tittonell P. (2024). Assessing Uncertainties and Discrepancies in Agricultural Greenhouse Gas Emissions Estimation in China: A Comprehensive Review. Environ. Impact Assess. Rev..

[13] Liu X., Wang S., Zhuang Q., Jin X., Bian Z., Zhou M., Meng Z., Han C., Guo X., Jin W. (2022). A Review on Carbon Source and Sink in Arable Land Ecosystems. Land.

[14] Li M., Peng J., Lu Z., Zhu P. (2023). Research Progress on Carbon Sources and Sinks of Farmland Ecosystems. Resour. Environ. Sustain..

[15] Chen C., Song M. (2019). Visualizing a Field of Research: A Methodology of Systematic Scientometric Reviews. PLoS ONE.

[16] Chen C. (2006). CiteSpace II: Detecting and Visualizing Emerging Trends and Transient Patterns in Scientific Literature. J. Am. Soc. Inf. Sci. Technol..

[17] Van Eck N.J., Waltman L. (2010). Software Survey: VOSviewer, a Computer Program for Bibliometric Mapping. Scientometrics.

[18] Zheng X., Lu Y., Yuan J., Baninla Y., Zhang S., Stenseth N.C., Hessen D.O., Tian H., Obersteiner M., Chen D. (2020). Drivers of Change in China’s Energy-Related CO_2_ Emissions. Proc. Natl. Acad. Sci. USA.

[19] Ledford H. (2015). How to Solve the World’s Biggest Problems. Nature.

[20] Okamura K. (2019). Interdisciplinarity Revisited: Evidence for Research Impact and Dynamism. Palgrave Commun..

[21] Xie H., Wen Y., Choi Y., Zhang X. (2021). Global Trends on Food Security Research: A Bibliometric Analysis. Land.

[22] Don A., Steinberg B., Schöning I., Pritsch K., Joschko M., Gleixner G., Schulze E.-D. (2008). Organic Carbon Sequestration in Earthworm Burrows. Soil Biol. Biochem..

[23] Beillouin D., Corbeels M., Demenois J., Berre D., Boyer A., Fallot A., Feder F., Cardinael R. (2023). A Global Meta-Analysis of Soil Organic Carbon in the Anthropocene. Nat. Commun..

[24] Rumpel C., Kögel-Knabner I. (2011). Deep Soil Organic Matter—A Key but Poorly Understood Component of Terrestrial C Cycle. Plant Soil.

[25] Poeplau C., Don A., Vesterdal L., Leifeld J., Van Wesemael B., Schumacher J., Gensior A. (2011). Temporal Dynamics of Soil Organic Carbon after Land-Use Change in the Temperate Zone–Carbon Response Functions as a Model Approach. Glob. Change Biol..

[26] Feng W., Shi Z., Jiang J., Xia J., Liang J., Zhou J., Luo Y. (2016). Methodological Uncertainty in Estimating Carbon Turnover Times of Soil Fractions. Soil Biol. Biochem..

[27] Huang Y., Lu X., Shi Z., Lawrence D., Koven C.D., Xia J., Du Z., Kluzek E., Luo Y. (2018). Matrix Approach to Land Carbon Cycle Modeling: A Case Study with the Community Land Model. Glob. Change Biol..

[28] Six J., Bossuyt H., Degryze S., Denef K. (2004). A History of Research on the Link between (Micro)Aggregates, Soil Biota, and Soil Organic Matter Dynamics. Soil Tillage Res..

[29] Six J., Elliott E.T., Paustian K. (2000). Soil Macroaggregate Turnover and Microaggregate Formation: A Mechanism for C Sequestration under No-Tillage Agriculture. Soil Biol. Biochem..

[30] Christensen B.T. (2001). Physical Fractionation of Soil and Structural and Functional Complexity in Organic Matter Turnover. Eur. J. Soil Sci..

[31] Oglesby R.T., Christman R.F., Driver C.H. (1968). The Biotransformation of Lignin to Humus—Facts and Postulates. Adv. Appl. Microbiol..

[32] Liang C., Schimel J.P., Jastrow J.D. (2017). The Importance of Anabolism in Microbial Control over Soil Carbon Storage. Nat. Microbiol..

[33] Xiao K.Q., Zhao Y., Liang C., Zhao M., Moore O.W., Otero-Fariña A., Zhu Y.G., Johnson K., Peacock C.L. (2023). Introducing the Soil Mineral Carbon Pump. Nat. Rev. Earth Environ..

[34] Liao Q., Zhang X., Li Z., Pan G., Smith P., Jin Y., Wu X. (2009). Increase in Soil Organic Carbon Stock over the Last Two Decades in China’s Jiangsu Province. Glob. Change Biol..

[35] Triberti L., Nastri A., Giordani G., Comellini F., Baldoni G., Toderi G. (2008). Can Mineral and Organic Fertilization Help Sequestrate Carbon Dioxide in Cropland?. Eur. J. Agron..

[36] Greene L.A. (2000). United Nations Framework Convention on Climate Change. Environ. Health Perspect..

[37] Forte A., Fagnano M., Fierro A. (2017). Potential Role of Compost and Green Manure Amendment to Mitigate Soil GHGs Emissions in Mediterranean Drip Irrigated Maize Production Systems. J. Environ. Manag..

[38] Roger C., Bowman M., McFadden J., Smith D., Wallander S. (2018). Tillage Intensity and Conservation Cropping in the United States.

[39] Sun X., Qian L., Cao Y., Wang M., Li N., Pang R., Si T., Yu X., Zhang X., Zuza E.J. (2024). Exploration of the Optimal Low-Carbon Peanut Rotation System in South China. Agric. Syst..

[40] Bhattacharyya P., Roy K.S., Neogi S., Adhya T.K., Rao K.S., Manna M.C. (2012). Effects of Rice Straw and Nitrogen Fertilization on Greenhouse Gas Emissions and Carbon Storage in Tropical Flooded Soil Planted with Rice. Soil Tillage Res..

[41] Lugato E., Bampa F., Panagos P., Montanarella L., Jones A. (2014). Potential Carbon Sequestration of European Arable Soils Estimated by Modelling a Comprehensive Set of Management Practices. Glob. Change Biol..

[42] Benites J.R., Derpsch R., McGarry D. The current Status and Future Growth Potential of Conservation Agriculture in the World Context. Proceedings of the “Soil Management for Sustainability”, 16th ISTRO Conference.

[43] Corbeels M., Cardinael R., Naudin K., Guibert H., Torquebiau E. (2019). The 4 per 1000 Goal and Soil Carbon Storage under Agroforestry and Conservation Agriculture Systems in Sub-Saharan Africa. Soil Tillage Res..

[44] Kassam A., Friedrich T., Derpsch R. (2019). Global Spread of Conservation Agriculture. Int. J. Environ. Stud..

[45] Friedrich T., Derpsch R., Kassam A. (2012). Overview of the Global Spread of Conservation Agriculture. Field Actions Sci. Rep..

[46] Ryan J., Rashid A., Torrent J., Yau S.K., Ibrikci H., Sommer R., Erenoglu E.B. (2013). Micronutrient Constraints to Crop Production in the Middle East-West Asia Region: Significance, Research, and Management. Adv. Agron..

[47] Allmaras R.R., Schomberg H.H. (1999). Conservation Tillage Unforeseen Advantage. Resour. Eng. Technol. Sustain. World.

[48] Follett R.F. (2001). Soil Management Concepts and Carbon Sequestration in Cropland Soils. Soil Tillage Res..

[49] Tian J., Dungait J.A.J., Hou R., Deng Y., Hartley I.P., Yang Y., Kuzyakov Y., Zhang F., Cotrufo M.F., Zhou J. (2024). Microbially Mediated Mechanisms Underlie Soil Carbon Accrual by Conservation Agriculture under Decade-Long Warming. Nat. Commun..

[50] Pumpanen J., Kolari P., Ilvesniemi H., Minkkinen K., Vesala T., Niinistö S., Lohila A., Larmola T., Morero M., Pihlatie M. (2004). Comparison of Different Chamber Techniques for Measuring Soil CO_2_ Efflux. Agric. For. Meteorol..

[51] Li H., Zhang H.B., Li G.C. (2020). Evaluation Method of Farmland Carbon Sequestration and Emission Reduction Technology.

[52] Chi J., Waldo S., Pressley S., O’Keeffe P., Huggins D., Stöckle C., Pan W.L., Brooks E., Lamb B. (2016). Assessing Carbon and Water Dynamics of No-till and Conventional Tillage Cropping Systems in the Inland Pacific Northwest US Using the Eddy Covariance Method. Agric. For. Meteorol..

[53] Yuan Y., Dai Q.D., Wang M.W. (2018). Greenhouse Gas Fluxes Dataset Effected by Land-Use Conversion from Double Rice Cropping to Vegetables in Southern China. J. Glob. Change Data Discov..

[54] Baldocchi D., Falge E., Gu L., Olson R., Hollinger D., Running S., Anthoni P., Bernhofer C., Davis K., Evans R. (2001). FLUXNET: A New Tool to Study the Temporal and Spatial Variability of Ecosystem–Scale Carbon Dioxide, Water Vapor, and Energy Flux Densities. Bull. Am. Meteorol. Soc..

[55] Xiao J., Sun G., Chen J., Chen H., Chen S., Dong G., Gao S., Guo H., Guo J., Han S. (2013). Carbon Fluxes, Evapotranspiration, and Water Use Efficiency of Terrestrial Ecosystems in China. Agric. For. Meteorol..

[56] Wu X., Wang S., Fu B., Liu Y., Zhu Y. (2018). Land Use Optimization Based on Ecosystem Service Assessment: A Case Study in the Yanhe Watershed. Land Use Policy.

[57] Zhang Z., Shen Z., Liu L., Zhang Y., Yu C., Cui L., Gao Y. (2023). Integrating Ecosystem Services Conservation into the Optimization of Urban Planning Policies in Eco-Fragile Areas: A Scenario-Based Case Study. Cities.

[58] Xie G.D., Xiao Y. (2013). Review of agro-ecosystem services and their values. Chin. J. Eco-Agric..

[59] Rodríguez J.P., Beard T.D., Bennett E.M., Cumming G.S., Cork S.J., Agard J., Dobson A.P., Peterson G.D. (2006). Trade-Offs across Space, Time, and Ecosystem Services. Ecol. Soc..

[60] Liu Q., Sun X., Wu W., Liu Z., Fang G., Yang P. (2022). Agroecosystem Services: A Review of Concepts, Indicators, Assessment Methods and Future Research Perspectives. Ecol. Indic..

[61] Bennett E.M., Peterson G.D., Gordon L.J. (2009). Understanding Relationships among Multiple Ecosystem Services. Ecol. Lett..

[62] Kremen C., Williams N.M., Bugg R.L., Fay J.P., Thorp R.W. (2004). The Area Requirements of an Ecosystem Service: Crop Pollination by Native Bee Communities in California. Ecol. Lett..

[63] Schipanski M.E., Barbercheck M., Douglas M.R., Finney D.M., Haider K., Kaye J.P., Kemanian A.R., Mortensen D.A., Ryan M.R., Tooker J. (2014). A Framework for Evaluating Ecosystem Services Provided by Cover Crops in Agroecosystems. Agric. Syst..

[64] Zhao M.Y., Liu Y.X., Zhang X.Y. (2022). A Review of Research Advances on Carbon Sinks in Farmland Ecosystems. Acta Ecol. Sin..

[65] Huang Y., Tang Y. (2010). An estimate of greenhouse gas (N_2_O and CO_2_) mitigation potential under various scenarios of nitrogen use efficiency in Chinese Croplands. Glob. Change Biol..

[66] Qian H., Zhu X., Huang S., Linquist B., Kuzyakov Y., Wassmann R., Minamikawa K., Martinez-Eixarch M., Yan X., Zhou F. (2023). Greenhouse Gas Emissions and Mitigation in Rice Agriculture. Nat. Rev. Earth Environ..

[67] Tangen B.A., Finocchiaro R.G., Gleason R.A. (2015). Effects of Land Use on Greenhouse Gas Fluxes and Soil Properties of Wetland Catchments in the Prairie Pothole Region of North America. Sci. Total Environ..

[68] Chen X., Ma C., Zhou H., Liu Y., Huang X., Wang M., Cai Y., Su D., Muneer M.A., Guo M. (2021). Identifying the Main Crops and Key Factors Determining the Carbon Footprint of Crop Production in China, 2001–2018. Resour. Conserv. Recycl..

[69] Mummey D.L., Smith J.L., Bluhm G. (1998). Assessment of Alternative Soil Management Practices on N_2_O Emissions from US Agriculture. Agric. Ecosyst. Environ..

[70] Chen B., Lu Q., Wei L., Fu W., Wei Z., Tian S. (2024). Global Predictions of Topsoil Organic Carbon Stocks under Changing Climate in the 21st Century. Sci. Total Environ..

[71] Mishra U., Hugelius G., Shelef E., Yang Y., Strauss J., Lupachev A., Harden J.W., Jastrow J.D., Ping C.L., Riley W.J. (2021). Spatial Heterogeneity and Environmental Predictors of Permafrost Region Soil Organic Carbon Stocks. Sci. Adv..

[72] Li J., Pei J., Pendall E., Fang C., Nie M. (2020). Spatial Heterogeneity of Temperature Sensitivity of Soil Respiration: A Global Analysis of Field Observations. Soil Biol. Biochem..

[73] Wiesmeier M., Urbanski L., Hobley E., Lang B., von Lützow M., Marin-Spiotta E., van Wesemael B., Rabot E., Ließ M., Garcia-Franco N. (2019). Soil Organic Carbon Storage as a Key Function of Soils-A Review of Drivers and Indicators at Various Scales. Geoderma.

[74] Conant R.T., Ryan M.G., Ågren G.I., Birge H.E., Davidson E.A., Eliasson P.E., Evans S.E., Frey S.D., Giardina C.P., Hopkins F.M. (2011). Temperature and Soil Organic Matter Decomposition Rates–Synthesis of Current Knowledge and a Way Forward. Glob. Change Biol..

[75] Smith P., Chapman S.J., Scott W.A., Black H.I.J., Wattenbach M., Milne R., Campbell C.D., Lilly A., Ostle N., Levy P.E. (2007). Climate Change Cannot Be Entirely Responsible for Soil Carbon Loss Observed in England and Wales, 1978–2003. Glob. Change Biol..

[76] Amundson R., Berhe A.A., Hopmans J.W., Olson C., Sztein A.E., Sparks D.L. (2015). Soil and Human Security in the 21st Century. Science.

[77] Zhou J., Wang Y., Tong Y., Sun H., Zhao Y., Zhang P. (2023). Regional Spatial Variability of Soil Organic Carbon in 0-5 m Depth and Its Dominant Factors. CATENA.

[78] Crowther T.W., Todd-Brown K.E.O., Rowe C.W., Wieder W.R., Carey J.C., Machmuller M.B., Snoek B.L., Fang S., Zhou G., Allison S.D. (2016). Quantifying Global Soil Carbon Losses in Response to Warming. Nature.

[79] Huo Y., Mi G., Zhu M., Chen S., Li J., Hao Z., Cai D., Zhang F. (2024). Carbon Footprint of Farming Practices in Farmland Ecosystems on the North and Northeast China Plains. J. Environ. Manag..

[80] Wang Q., Zhou F., Shang Z., Ciais P., Winiwarter W., Jackson R.B., Tubiello F.N., Janssens-Maenhout G., Tian H., Cui X. (2020). Data-Driven Estimates of Global Nitrous Oxide Emissions from Croplands. Natl. Sci. Rev..

[81] Jiang Y., Carrijo D., Huang S., Chen J., Balaine N., Zhang W., van Groenigen K.J., Linquist B. (2019). Water Management to Mitigate the Global Warming Potential of Rice Systems: A Global Meta-Analysis. Field Crops Res..

[82] Kremen C., Miles A. (2012). Ecosystem Services in Biologically Diversified versus Conventional Farming Systems: Benefits, Externalities, and Trade-Offs. Ecol. Soc..

[83] Zhang Y., Huang K., Yu Y., Yang B. (2017). Mapping of Water Footprint Research: A Bibliometric Analysis during 2006–2015. J. Clean. Prod..

[84] Chen C. (2004). Searching for Intellectual Turning Points: Progressive Knowledge Domain Visualization. Proc. Natl. Acad. Sci. USA.

[85] Fang J.Y., Guo Z.D., Piao S.L., Chen A.P. (2007). Terrestrial vegetation carbon sinks in China, 1981-2000. Sci. China Ser. D Earth Sci..

[86] Pacala S.W., Hurtt G.C., Baker D., Peylin P., Houghton R.A., Birdsey R.A., Heath L., Sundquist E.T., Stallard R.F., Ciais P. (2001). Consistent Land and Atmosphere-Based U.S. Carbon Sink Estimates. Science.

[87] Yu G.R., He N.P., Wang Q.F. (2013). Theoretical Basis and Comprehensive Assessment: Theoretical Basis and Comprehensive Assessment.

[88] Ye F., Wang L., Razzaq A., Tong T., Zhang Q., Abbas A. (2023). Policy Impacts of High-Standard Farmland Construction on Agricultural Sustainability: Total Factor Productivity-Based Analysis. Land.

[89] Zhang W., Furtado K., Wu P., Zhou T., Chadwick R., Marzin C., Rostron J., Sexton D. (2021). Increasing Precipitation Variability on Daily-to-Multiyear Time Scales in a Warmer World. Sci. Adv..

[90] Velimirovic A., Jovovic Z., Przulj N. (2021). From Neolithic to Late Modern Period: Brief History of Wheat. Genetika.

